# Sex-Related Disparities in the Incidence and Outcomes of Community-Acquired Pneumonia among Type 2 Diabetes Patients: A Propensity Score-Matching Analysis Using the Spanish National Hospital Discharge Database for the Period 2016–2019

**DOI:** 10.3390/jcm10173975

**Published:** 2021-09-02

**Authors:** Ana Lopez-de-Andres, Rodrigo Jimenez-Garcia, Valentin Hernandez-Barrera, Javier de Miguel-Diez, Jose M. de Miguel-Yanes, David Carabantes-Alarcon, Jose J. Zamorano-Leon, Sara Sanz-Rojo, Marta Lopez-Herranz

**Affiliations:** 1 Department of Public Health & Maternal and Child Health, Faculty of Medicine, Universidad Complutense de Madrid, 28040 Madrid, Spain; anailo04@ucm.es (A.L.-d.-A.); dcaraban@ucm.es (D.C.-A.); jjzamorano@ucm.es (J.J.Z.-L.); ssanz01@ucm.es (S.S.-R.); 2 Preventive Medicine and Public Health Teaching and Research Unit, Health Sciences Faculty, Universidad Rey Juan Carlos, Alcorcón, 28922 Madrid, Spain; valentin.hernandez@urjc.es; 3 Respiratory Department, Hospital General Universitario Gregorio Marañón, Instituto de Investigación Sanitaria Gregorio Marañón, Universidad Complutense de Madrid, 28040 Madrid, Spain; javier.miguel@salud.madrid.org; 4 Internal Medicine Department, Hospital General Universitario Gregorio Marañón, Instituto de Investigación Sanitaria Gregorio Marañón, Universidad Complutense de Madrid, 28040 Madrid, Spain; josemaria.demiguel@salud.madrid.org; 5 Nursing Department, Faculty of Nursing, Physiotherapy and Podology, Universidad Complutense de Madrid, 28040 Madrid, Spain; martal11.ucm@gmail.com

**Keywords:** community-acquired pneumonia, type 2 diabetes mellitus, hospitalizations, outcomes

## Abstract

(1) Background: To analyze incidence, clinical characteristics, procedures, and in-hospital outcomes among patients hospitalized with community-acquired pneumonia (CAP) according to the presence of T2DM in Spain (2016–2019) and to assess the role of gender among those with T2DM. (2) Methods: Using the Spanish National Hospital Discharge Database, we estimated hospitalized CAP incidence. Propensity score matching was used to compare population subgroups. (3) Results: CAP was coded in 520,723 patients, of whom 140,410 (26.96%) had T2DM. The hospitalized CAP incidence was higher in patients with T2DM (both sexes) (IRR 4.25; 95% CI 4.23–4.28). The hospitalized CAP incidence was higher in men with T2DM than in women with T2DM (IRR 1.46; 95% CI 1.45–1.47). The hospitalized CAP incidence among T2DM patients increased over time; however, the in-hospital mortality (IHM) decreased between 2016 and 2019. IHM was higher among non-T2DM men and women than matched T2DM men and women (14.23% and 14.22% vs. 12.13% and 12.91%; all *p* < 0.001, respectively), After adjusting for confounders, men with T2DM had a 6% higher mortality risk than women (OR 1.06; 95% CI 1.02–1.1). (4) Conclusions: T2DM is associated with a higher hospitalized CAP incidence and is increasing overtime. Patients hospitalized with CAP and T2DM have lower IHM. Male sex is a significant risk factor for mortality after CAP among T2DM patients.

## 1. Introduction

People with type 2 diabetes mellitus (T2DM) are at greater risk of infections, showing worse infection outcomes than those without diabetes [1]. Community-acquired pneumonia (CAP) is an infection that shows an increase in incidence over time and is highly influenced by age and comorbidities [2,3]. CAP often requires hospital admission, especially among older adults [4,5]. Campling et al. [6] found that patients with T2DM have a significantly higher risk of hospital admission for CAP (OR 1.18; 95% CI 1.13–1.23). In Spain, approximately 12% of patients with T2DM admitted to hospital with a first diagnosis of pneumonia die in hospital [7].

The hospitalized CAP incidence in patients with and without T2DM is increasing [8,9,10]. In Spain, a previous population-based cohort study found that the incidence in patients with T2DM increased significantly (IRR 1.27; 95% CI 1.23–1.31) between 2004 and 2013. Furthermore, the incidence in patients with T2DM was significantly higher than in subjects without diabetes (IRR 1.05; 95% CI 1.03–1.07) [10].

Sex differences may play an active role in the incidence and outcomes among patients with CAP. Several studies indicate that male sex is a significant risk factor for mortality after CAP [11,12]; however, other authors conclude that male sex is not a significant risk factor for CAP [13]. Results have been described in patients with diabetes that are similar to results found in the general population [7,10]; however, data regarding the results of hospitalization after CAP among men and women with diabetes are scarce.

Given these inconclusive findings, we used administrative data from an entire country over a four-year period to compare incidence, clinical characteristics, use of therapeutic procedures, and in-hospital outcomes in patients hospitalized with CAP divided by T2DM status and gender. We used propensity score matching (PSM) to compare CAP-associated hospital outcomes between men and women with and without T2DM, and between men and women with T2DM. Finally, we identified the variables associated with in-hospital mortality (IHM) for patients with T2DM according to sex.

## 2. Materials and Methods

### 2.1. Study Design, Study Population and Data Assessment

To achieve the established objectives, a cohort study was carried out based on hospital discharge reports collected through the Hospital Discharge Records of the Spanish National Health System (RAE-CMBD, Registro de Actividad de Atención Especializada. Conjunto Mínimo Básico de Datos (Registry of Specialized Health Care Activities. Minimum Basic Data Set)) for the period running from 1 January 2016 to 31 December 2019. The discharge records are coded based on the International Classification of Disease, Tenth Revision (ICD-10). Details on RAE-CMBD are available online [14].

We selected patients aged ≥18 years with a primary diagnosis of CAP using the specific diagnosis assigned according to ICD-10 codes (Supplementary Table S1) recorded in the discharge records. 

The population was divided according to sex and to the presence of T2DM. Subjects with a diagnosis code for T2DM (E11.x) in any diagnosis field were classified as having T2DM. Patients with a code for type 1 diabetes mellitus (T1DM) (E10.x) in any diagnosis field were excluded. 

The main study variables were trends in the hospitalized CAP incidence among men and women with and without T2DM and the IHM and length of hospital stay (LOHS) in these subgroups. We also analyzed comorbidities and therapeutic procedures.

We calculated incidence rates of admission for CAP hospitalization per 10,000 inhabitants with and without T2DM for the period 2016–2019. The estimated Spanish population suffering with T2DM was obtained using data from the 2016/17 Spanish National Health Survey (SNHS2017) [15]. This database can be downloaded free of charge from the Spanish National Statistics Institute webpage [15]. In the SNHS2017 database, 23,090 adults aged ≥15 years were interviewed. Using the SNHS2017 database, we obtained the estimated prevalence of T2DM for men and women in the age groups used in our investigation (18–49 years, 50–64 years, 65–79 years, and ≥80 years). These specific sex-age group prevalence of T2DM were then multiplied by the population living in Spain on 1 July for each year studied in each of these sex and age strata. The census populations according to age and sex can be freely downloaded from the Spanish National Statistics Institute’s webpage [16]. We then divided the amount of CAP hospitalization among T2DM patients in each sex and age group by the estimated number of subjects suffering from T2DM living in Spain in the same sex–age groups to obtain the incidence of CAP hospitalization per 10,000 persons with T2DM. The same method was used to calculate the sex and age group incidences among those without T2DM. 

The patient-level variables analyzed included age and sex. Comorbidity was quantified using the Charlson Comorbidity Index (CCI) calculated based on ICD-10 codes, as described elsewhere [17,18]. We then used CCI in two different ways. First, we used CCI as a continuous variable by adding the total number of conditions included in the CCI codified in each patient. The mean value of CCI was then calculated and used to provide a measure of the comorbidity present in the different study populations along the study period. Second, each of the individual conditions included in the CCI were used to conduct the propensity score matching and to assess their possible association with in-hospital mortality after CAP among men and women.

Concerning the procedures, we studied mechanical ventilation (non-invasive and invasive) (see ICD-10 codes in Supplementary Table S1).

Regarding the detection of pathogens among patients with pneumonia, we only identified those coded and confirmed by a laboratory, including *Aspergillus*, *Candidiasis*, *Escherichia coli*, *Haemophilus influenzae*, *Klebsiella pneumoniae*, *Legionella*, non-specified *Streptococcus*, other Gram-negative bacteria, *Pseudomonas aeruginosa*, *Staphylococcus aureus*, *Streptococcus pneumonia*, *Influenza virus,* and other viruses (See ICD-10 codes in Supplementary Table S1).

We used a PSM method to create subpopulations that were comparable based on their baseline conditions [19]. We performed three PSM analyses, namely, men with T2DM and non-T2DM men, women with T2DM and non-T2DM women, and T2DM men and T2DM women. The PSM was conducted using multivariable logistic regression in which the matching variables were age, sex, and comorbid conditions present at admission. These methods have been described in detail elsewhere [7].

### 2.2. Statistical Analysis

Incidence was analyzed using Poisson regression models adjusted for age and sex when required, providing incidence rate ratios (IRR) with 95% confidence intervals (95% CI). 

Descriptive statistical analysis included mean and standard deviation (SD) or median and interquartile range (IQR) for continuous variables and frequency and percentage for categorical variables.

Continuous variables were compared using the t-test or Mann-Whitney test. Categorical variables were compared using the Chi-square test.

McNemar’s test and a paired *t*-test were used to compare the study subgroups after PSM.

To identify which variables were independently associated with IHM, we conducted multivariable logistic regression. We constructed models separately for men and women and according to T2DM status. Finally, using the entire database, we analyzed the effect of sex. To construct these models, the following steps were performed: (i) univariable analysis of each variable; (ii) selection of the variables to be included in the multivariable analysis, in which we included all variables with a significant association (*p* < 0.10) in the univariable test and those identified as important in the literature search; (iii) verification of the importance of each variable included in the model using the Wald statistic and the comparison of the successive models to the previous models using the Likelihood Ratio test; and (iv) after the model was obtained, the analysis of the possible linearity between variables and determination of interactions. Estimates were the odds ratios (ORs) with their 95% CIs.

The statistical analysis and PSM were conducted using Stata version 14 (Stata, College Station, TX, USA), and significance was set at *p* < 0.05 (two-sided).

### 2.3. Ethics

The RAE-CMBD is owned by the Spanish Ministry of Health and can be accessed upon request [20]. Given the characteristics of this registry, which is anonymous, it does not require individual written consent from the patients or ethics committee approval according to Spanish legislation.

## 3. Results

A total of 520,723 patients (58.91% men and 41.09% women) aged ≥18 years were hospitalized with a primary diagnosis of CAP in Spain during the period 2016–2019. T2DM was diagnosed in 140,410 patients (26.96%). The prevalence of T2DM was higher among men than among women (28.32% vs. 25.01%; *p* < 0.001).

### 3.1. Incidence of Patients Admitted to Hospitals with a Diagnosis of CAP According to T2DM Status

As can been seen in Table 1, among patients with T2DM, we found that the hospitalized CAP incidence coding increased significantly from 92.03 in 2016 to 125.51 in 2019 cases per 10,000 persons with T2DM (*p* < 0.001). In patients without T2DM, the incidence of admissions also increased significantly (*p* < 0.001) over the study period. The incidence was significantly higher in people with T2DM than in non-diabetic people for all years analyzed (*p* < 0.001). The Poisson regression model yield an adjusted IRR of 4.25 (95% CI 4.23–4.28) for hospitalized CAP incidence for T2DM subjects vs. non-T2DM subjects. The hospitalized CAP incidence coding increased significantly in both men and women with T2DM, from 108.33 cases per 10,000 men with T2DM men and 74.08 cases per 10,000 women with T2DM in 2016 to 147.08 and 101.25 in 2019, respectively (all *p* < 0.001). A significant increase in the figures for men and women without T2DM patients was also found (Table 1). 

Age and CCI increased significantly over time in both patients with and without T2DM (all *p* < 0.001). LOHS was around 7 days for all years analyzed and diabetes status. IHM decreased significantly from 12.73% and 12.63% in 2016 for patients with and without T2DM to 11.97% and 11.78% in 2019, respectively (Table 1).

Over time, we found that *Haemophilus influenzae*, *Influenza virus*, *Streptococcus pneumoniae*, and other viruses increased significantly over time in both patients with and without T2DM. Other Gram-negative bacteria increased significantly from 0.52% in 2016 to 0.66% in 2019 (*p* = 0.006) only in patients with T2DM, as can been seen in Supplementary Table S2.

### 3.2. Clinical Characteristics and Hospital Outcomes for Women and Men Admitted to Hospital with a Diagnosis of CAP According to T2DM Status

Table 2 shows incidence, clinical characteristics, therapeutic procedures, and hospital outcomes before and after PSM for women with CAP according to the presence of T2DM.

The incidence was significantly higher in women with T2DM than in those without T2DM (90.82 cases per 10,000 women with T2DM vs. 21.38 cases per 10,000 women without T2DM, *p* < 0.001). The corresponding adjusted IRR obtained with Poisson regression was 4.34 (95% CI 4.28–4.41).

The mean age was significantly higher among women with T2DM (80.18; SD = 10.7 years) than non-T2DM women (74.35; SD = 17.48 years), and women with T2DM also had a higher mean CCI and more specific chronic conditions. During hospitalization, women with T2DM received invasive mechanical ventilation (1.70%) significantly less often than women without T2DM (2.18%) (*p* < 0.001); however, women with T2DM more frequently received non-invasive mechanical ventilation (3.28% vs. 2.49%; *p* < 0.001). The median LOHS was 7 days for women with and without T2DM. The crude IHM was 12.91% for women with T2DM and 11.92% for non-T2DM women (*p* < 0.001).

After PSM, invasive mechanical ventilation continued to be less frequent and non-invasive ventilation continued to be more frequent among T2DM women. However, the IHM turn around and was lower (12.91%) for women with T2DM than for women without T2DM (14.22%: *p* < 0.001).

Incidence, clinical characteristics, therapeutic procedures, and hospital outcomes before and after PSM for men with and without T2DM hospitalized with CAP are shown in Table 3.

The crude hospitalized CAP incidence was significantly higher in men with T2DM than in non-diabetic men (132.92 cases per 10,000 men with T2DM vs. 31.33 cases per 10,000 men without T2DM; *p* < 0.001). The adjusted IRR estimated for men T2DM vs. men without T2DM was 4.18 (95% CI 4.12–4.24).

Before PSM, we found significant differences in the distribution of age and comorbidities (CCI) between men with and without T2DM, as was the case in women. However, men with T2DM had a higher prevalence of chronic obstructive pulmonary disease (COPD) than non-T2DM men (40.14% vs. 37%; *p* < 0.001). As in women, men with T2DM received more non-invasive mechanical ventilation and less invasive mechanical ventilation (all *p* < 0.001). LOHS was around 7 days in both men with and without T2DM. Non-T2DM men had higher crude IHM than men with T2DM (12.65% vs. 12.13%, *p* < 0.001).

After PSM, we found that among men with T2DM, invasive mechanical ventilation continued to be less frequent, non-invasive ventilation continued to be more frequent, and IHM continued to be lower than in non-T2DM men.

### 3.3. Incidence, Clinical Characteristics, and Hospital Outcomes for Diabetic Patients Admitted to Hospital with a Diagnosis of CAP According to Sex

As can be seen in Table 4, the incidence was significantly higher in men than in women with T2DM. The results of the Poisson regression model showed that the overall hospitalized CAP incidence over the period 2016–2019 was 1.46 times higher among men with T2DM than among women with T2DM (IRR 1.46; 95% CI 1.45–1.47).

When we compared T2DM men with T2DM women, we observed that men were younger (76.68 ± 10.56 years vs. 80.18 ± 10.79 years; *p* < 0.001), with a higher mean CCI (1.57 ± 1.12 vs. 1.28 ± 1.01). Men also more frequently had myocardial infarction, peripheral vascular disease, cerebrovascular disease, COPD, peptic ulcer, liver disease (mild and moderate/severe), renal disease, cancer, metastatic cancer, and acquired immune deficiency syndrome (AIDS). However, congestive heart failure, dementia, and rheumatoid disease were more prevalent in women than in men. 

After PSM, men with T2DM received non-invasive mechanical ventilation (2.84%) significantly less often than women with T2DM (3.28%) (*p* < 0.001); however, men with T2DM more frequently received invasive mechanical ventilation than women with T2DM (2.1% vs. 1.7%; *p* < 0.001). The difference in IHM was statistically significant (*p* = 0.006) after PSM, with proportions of 13.48% for men with T2DM and 12.91% for women with T2DM.

Regarding the pathogen’s isolation, after PSM, the prevalence of *Escherichia coli*, *Klebsiella pneumoniae*, *Legionella*, non-specified *Streptococcus*, other Gram-negative bacteria, and *Staphylococcus aureus* was significantly higher in men with T2DM than in women with T2DM. However, the prevalence of the *Influenza* virus (4.09% vs. 3.55%; *p* < 0.001) and other viruses (0.98% vs. 0.7%; *p* < 0.001) was higher among T2DM women (Supplementary Table S3).

### 3.4. Multivariable Analysis of Variables Associated with IHM among Diabetic Men and Women with CAP

As can been seen in Table 5, after multivariable adjustment (the results of the univariable analysis are shown in Supplementary Table S4), the risk of dying in hospital increased with age, myocardial infarction, congestive heart failure, cerebrovascular disease, dementia, hemiplegia or paraplegia, renal disease, cancer, moderate/severe liver disease, and metastatic cancer among men and women with T2DM. Peripheral vascular disease was associated with IHM in women with T2DM, but not in men.

In both men and women with T2DM, the presence of COPD reduced the IHM. The presence of mild liver disease reduced the IHM only in women with T2DM (OR 0.81; 95% CI 0.7–0.95).

The need for mechanical ventilation (non-invasive and invasive) during admission increased the risk of IHM in T2DM patients irrespective of gender (OR 2.47; 95% CI 2.26–2.7 and OR 8.08; 95% CI 7.318.93, respectively). 

Over time, the IHM decreased significantly in both men and women with T2DM. Finally, as found with the PSM, men with T2DM were significantly more likely to die in hospital than T2DM women (OR 1.06; 95% CI 1.02–1.1). 

## 4. Discussion

This nationwide registry and population-based observational cohort study showed that hospitalized CAP incidences were higher in patients with T2DM than in those without T2DM in all years analyzed. After PSM, invasive mechanical ventilation was used less frequently in T2DM patients than in non-T2DM patients and less frequently in T2DM women than in T2DM men. Non-invasive mechanical ventilation was used more frequently in T2DM patients and more frequently in T2DM women than in men. IHM was significantly lower in men and women with T2DM than in non-diabetic subjects. Mechanical ventilation (non-invasive and invasive) appeared to be associated with a higher IHM among T2DM patients. In the fully adjusted model, men with T2DM had a 6% higher risk of dying in hospital after CAP than women with T2DM. 

According to our database, the rates of hospitalization for CAP in patients with and without T2DM increased significantly from 2016 to 2019. This trend had already been demonstrated in the general population, suggesting that aging and more low-severity cases presenting at the emergency rooms could partly explain this increase [2,8,9]. Regarding patients with diabetes, in a previous study carried out including 223,715 patients with T2DM aged ≥40 years between 2004 and 2013 in Spain, the authors concluded that hospitalization rates increased from 812.64 to 923.26 cases per 100,000 inhabitants with an adjusted IRR for pneumonia of 1.66 (95% CI 1.65–1.67) from the first to the last year analyzed [10].

Recently, a meta-analysis designed to assess the association between T2DM and CAP revealed that patients with diabetes have a 1.64-times higher risk of CAP (RR 1.64; 95% CI 1.55–1.73) [21]. We found that the hospitalized CAP incidence was higher in patients with T2DM than in those without T2DM, irrespective of gender. This finding has been reported elsewhere in cohort and case-control studies [5,10,22].

As we expected, hospitalized CAP incidences were higher in T2DM men than in T2DM women. These results agree with data from general populations [2,23,24] where men have been identified as having a higher incidence of pneumonia and a worse prognosis [2,23,24]. 

The results of the present study are in line with those found in the literature, thereby demonstrating that patients with diabetes have a worse risk profile than patients without diabetes [25]. In addition, as expected, and consistent with other investigations, older age, myocardial infarction, congestive heart failure, cerebrovascular disease, dementia, hemiplegia or paraplegia, renal disease, cancer, moderate/severe liver disease, and metastatic cancer were risk factors for IHM [10].

The lower-than-expected mortality among T2DM men and women with concomitant COPD could be due to several factors. First, given the overlap in symptoms/clinical findings between COPD exacerbations and pneumonia, exacerbations could be mistakenly coded as CAP. This misclassification has been suggested by other authors when ICD10 codes are used [26]. Second, increased awareness of disease by both clinicians and patients may result in those patients with COPD being more likely to be hospitalized with less severe pneumonia. Third, this could also be due to a protective anti-inflammatory effect of inhaled corticosteroids and different immune responses secondary to an altered microbiome [27,28,29].

The results of the present study indicate that during admission for CAP, men and women with T2DM received non-invasive mechanical ventilation more frequently than matched non-T2DM men and women. The overuse of non-invasive mechanical ventilation among people with diabetes has been described previously [7,10]. In a study about mechanical ventilation use in 56,158 patients with CAP who received ventilator support, the authors found an increase in the prevalence of comorbidities over time that could partially explain the higher need for ventilatory support [30]. Furthermore, this procedure was more used in T2DM women than in T2DM men; however, invasive mechanical ventilation is less frequently used in diabetic women. The higher rates of non-invasive ventilation in women with T2DM may be explained by the fact that women with CAP have more hospital complications than men, and this may contribute to treatment decisions that involve a less invasive approach than providing invasive mechanical ventilation. As described in other studies, mechanical ventilation (non-invasive and invasive) were risk factors for IHM in both men and women with T2DM [7,10]. Previous studies have reported high IHM rates, ranging between 46% and 56%, among patients with CAP who required invasive mechanical ventilation [31,32,33]. Variables associated with a worse prognosis in these patients included advanced age, comorbidities, and a greater severity of pneumonia and organ dysfunction upon hospital admission [31,32,33]. Men and women with diabetes admitted with CAP had lower IHM than men and women without diabetes. This finding confirms those of previous research in Spain from 2004 to 2013, albeit without PSM, where diabetes was associated with a lower IHM (OR 0.92, 95% CI 0.91 to 0.94) after a CAP hospitalization [10]. These findings are very similar to the current data showing that it is possible that patients with diabetes are hospitalized with a less severe disease or that the presence of obesity could explain this lower mortality in patients with diabetes. Several studies have indicated that obesity was not associated with a higher mortality risk during admission for CAP [34,35,36]. A meta-analysis by Nie et al. concluded that overweight and obesity were significantly associated with reduced risk of pneumonia mortality (RR 0.83, 95% CI 0.77 to 0.91, *p* < 0.01) and suggested that an ‘obesity survival paradox’ exists for pneumonia [34].

We found that male sex was a risk factor for mortality in T2DM patients with CAP. In a recent study, the authors found that 30-day mortality was 19% higher in men than in women (OR 1.19; 95% CI 1.06–1.34) [24]; however, Arnold et al. [37] found that women have worse outcomes for CAP with a 28-day mortality OR of 1.15 (95% CI 1.02 to 1.30). 

Overall, it seems that males develop respiratory tract infections more frequently and more severely than females [38,39]. The reason for this finding is probably multifactorial [38,39]. Among the different hypotheses that could, at least in part, explain these differences are (i) biological differences, mainly due to sex hormones. In general, estrogens at physiological concentrations are thought to play an immune-stimulating role by upregulating both cellular and humoral immunity; (ii) anatomic differences of the respiratory tract; (iii) lifestyle factors, such as smoking or alcohol consumption, habits that are more common in males; (iv) socially defined sex roles. The perception of disease severity is different between males and females, and this can result in an earlier detection among women; and (v) on the other hand, a gender-related bias in the provision of care and the use of hospital resources has been reported among women with CAP, resulting in delayed hospital admission and, consequently, necessary care [38,39].

Finally, regarding CAP pathogens, the more impressive result was the year-by-year increase in *Streptococcus pneumoniae* frequency. 

In a recent review, it was found that Spain reported the highest incidence rates for hospitalized pneumococcal CAP in Europe [40]. De Miguel et al. analyzed national laboratory data from invasive pneumococcal disease (IPD) cases that affected populations during 2009–2019. In adults, the burden of disease according to all serotypes remained constant over time for the age group of 18–64 years and was moderately increased for adults aged ≥65 years [41]. However, vaccination with the pneumococcal conjugate vaccine for 13 serotypes (PCV13) in children, due to herd protection, seems to control IPD cases caused by the serotypes included in this vaccine. However, these authors recommend interpreting the results with caution in the context of low vaccine coverage in Spanish adults aged ≥65 years. In Spain, since 2004, the recommendation by the Ministry of Health is the use of a polysaccharide vaccine that contains 23 serotypes (PPV23) for all persons aged ≥65 years. However, since 2016, several regions have introduced PCV13 for adults [42]. The latest coverages reported by the public health authorities were 8% for PCV13 regions and 25% for PPV23 regions in 2017 vs. 22% for PCV13 regions and 26% for PPV23 regions in 2018, without information for 2019 [41]. 

Spanish authors have suggested that, besides year-to-year variability, the increase in bacterial and viral isolations over time might reflect a changing epidemiology due to a potential increase in patients with worse socio-sanitary conditions or with previous antimicrobial therapy and to an improvement over time in microbiological surveillance with a higher proportion of patients with samples being collected and improved microbiological diagnostic technologies [40,41,43]. Further evaluation of the impact of pneumococcal vaccination on adult CAP is necessary in our country. 

The strength of our findings lies in the large sample size with data from over 520,723 episodes of CAP (26.96% with T2DM), the widespread coverage of the population of an entire country (> 95% of all hospital admissions), the standardized methodology (which has been extensively used for research in Spain [2,3,7,10,30]), and the reliability of diabetes and CAP coding in the RAE-CMBD [44]. Nevertheless, we should point out several limitations. First, the use of administrative claims data based on ICD coding to identify patients with pneumonia is not as robust as prospective clinical studies, and this must be considered because incidences are frequently underestimated [45,46]. However, McLaughlin et al. conducted a systematic literature review of the hospitalized CAP incidence among US adults, finding that administrative claims databases were the most common data source [46]. Previous studies have assessed the validity of discharge diagnosis codes for pneumonia [47,48]. Using ICD9 coding, Garde et al. reported that the overall sensitivity was 72.4% when pneumonia was the primary code and 79.5% for combined primary and secondary codes [47]. Therefore, hospital administrative data may leave a quarter of pneumonia cases undetected [47]. Yu et al. observed that the validity of coding was affected by age with a higher sensitivity among those aged ≥65 years than among those aged 18–64 years (65% vs. 63%) and a better specificity among the younger group (93% vs. 85%) [48]. A very recent review on the validity of 13 studies identifying pneumonia cases concluded that sensitivities ranged from 31.3% to 97.8% (median, 65.1%; IQR 52.5–72.4) [49].

In our study, we used data based on ICD10. Previous studies have reported a modest undercount of all-cause pneumonia in the ICD-10-CM compared to the ICD-9-CM for adults [26]. Skull et al. compared ICD10 codes against medical record notations of pneumonia, reporting very high sensitivity (97.8%; 95% CI 97.1–98.3) and specificity (96.9%; 95% CI 96.2–97.5) [50]. However, as this study was conducted in only two large tertiary Australian hospitals, the results cannot be generalized to other countries. Further studies are needed to assess the accuracy of hospital discharge administrative databases for pneumonia in Spain and elsewhere. Second, our data source was an administrative database supported by the information that physicians recorded in discharge reports; therefore, the database lacks information on clinical characteristics, glycemic control, medical treatments, and the duration of T2DM. Furthermore, data other than those included in the ICD-10 coding on duration of ventilatory support, days in the intensive care unit, vaccinations, or severity of the respiratory disease were not available. Third, while the PSM process helped to attenuate differences in baseline characteristics and clinical variables, complete elimination of residual confounding is difficult to achieve in observational studies. Fourth, anonymity precludes the extraction of specific data that may affect the results (i.e., people who moved from one hospital to another could appear twice). Fifth, we have not analyzed data on smoking status and obesity, which could also affect risk and outcomes of CAP. We have not included these variables because according to the RAE-CMBD methodology in the secondary diagnosis fields (from 2 to 20), only those diagnoses that have induced the use of additional therapeutic or diagnosis procedures during the hospital admission or have negatively affected the LOHS or the IHM should be recorded [14]. In our opinion, smoking or obesity possibly do not affect the clinical course of most patients suffering from CAP and would therefore be under-codified, providing a false image of the study populations. Future investigations based on reliable data should include these conditions. Sixth, obesity is frequent among T2DM patients, and, due to the limitations of chest radiography and the frequent presence of comorbid conditions in obese individuals, the diagnosis of CAP can be challenging among these patients.

## 5. Conclusions

In conclusion, T2DM is associated with a higher hospitalized CAP incidence and is increasing over time. Patients hospitalized with CAP and T2DM have lower IHM than patients without T2DM, irrespective of gender. Male sex is a significant risk factor for mortality after CAP among T2DM patients. Our findings should be taken into consideration when planning future actions to improve the treatment and care that T2DM patients hospitalized with CAP receive. 

## Figures and Tables

**Table 1:** Incidence, clinical characteristics, and in-hospital outcomes of patients hospitalized with community-acquired pneumonia (CAP) in Spain from 2016 to 2019 according to the presence of T2DM.

Variables	2016		2017		2018		2019		*p*-Value	
	T2DM	No T2DM	T2DM	No T2DM	T2DM	No T2DM	T2DM	No T2DM	T2DM	No T2DM
*N*, (Incidence per 10,000 subjects)	29,135 (92.03)	85,652 (23.86)	34,564 (109.18)	91,787 (25.56)	38,939 (126.11)	103,298 (28.35)	37,772 (125.51)	99,576 (26.94)	<0.001	<0.001
*N*, (Incidence per 10,000 men)	17,973 (108.33)	50,599 (29.15)	21,425 (129.13)	53,179 (30.63)	24,072 (148.02)	59,121 (33.58)	23,434 (147.08)	56,987 (31.92)	<0.001	<0.001
*N*, (Incidence per 10,000 women)	11,162 (74.08)	35,053 (18.9)	13,139 (87.21)	38,608 (20.82)	14,867 (101.73)	44,177 (23.47)	14,338 (101.25)	42,589 (22.29)	<0.001	<0.001
Age, mean (SD)	77.5 (10.86)	72.28 (17.18)	78.25 (10.5)	73.65 (16.58)	78.12 (10.72)	73.21 (16.77)	78.08 (10.91)	72.98 (17.04)	<0.001	<0.001
18–49 years, *n* (%)	498 (1.71)	10,648 (12.43)	415 (1.2)	9594 (10.45)	498 (1.28)	11,309 (10.95)	523 (1.38)	11,252 (11.3)	0.007	<0.001
50–64 years, *n* (%)	3154 (10.83)	13,608 (15.89)	3384 (9.79)	13,881 (15.12)	4034 (10.36)	16,374 (15.85)	3972 (10.52)	16,429 (16.5)	0.900	<0.001
65–79 years, *n* (%)	10,853 (37.25)	23,697 (27.67)	12,460 (36.05)	24,924 (27.15)	14,003 (35.96)	28,231 (27.33)	13,820 (36.59)	27,050 (27.17)	0.235	0.094
≥80 years, *n* (%)	14,630 (50.21)	37,699 (44.01)	18,305 (52.96)	43,388 (47.27)	20,404 (52.4)	47,384 (45.87)	19,457 (51.51)	44,845 (45.04)	0.136	0.210
CCI index, mean (SD)	1.42 (1.07)	1.09 (0.99)	1.44 (1.08)	1.15 (1)	1.45 (1.09)	1.13 (1.02)	1.51 (1.11)	1.17 (1.03)	<0.001	<0.001
LOHS, Median (IQR)	7 (8)	8 (7)	7 (7)	7 (7)	7 (7)	7 (6)	7 (7)	7 (6)	0.554	0.766
IHM, *n* (%)	3708 (12.73)	10,814 (12.63)	4416 (12.78)	11,585 (12.62)	4806 (12.34)	12,811 (12.4)	4522 (11.97)	11,726 (11.78)	0.003	<0.001

T2DM: type 2 diabetes mellitus; CCI: Charlson comorbidity index; LOHS: length of hospital stay; IHM: in-hospital mortality; SD: standard deviation; IQR: interquartile range. *p* value for time trend.

**Table 2:** Distribution of study covariates and hospital outcomes of women with and without T2DM hospitalized with community-acquired pneumonia (CAP) in Spain (2016–2019), before and after propensity score matching (PSM).

	Before PSM			After PSM		
	T2DM	No T2DM	*p*-Value	T2DM	No T2DM	*p*-Value
*N* (incidence per 10,000 women)	53,506 (90.82)	160,427 (21.38)	<0.001	-	-	-
Age, mean (SD)	80.18 (10.7)	74.35 (17.48)	<0.001	80.18 (10.7)	80.60 (10.89)	<0.001
18–49, *n* (%)	643 (1.2)	17,951 (11.19)	<0.001	643 (1.2)	692 (1.29)	0.177
50–64, *n* (%)	4420 (8.26)	23,707 (14.78)	<0.001	4420 (8.26)	4308 (8.05)	0.211
65–79, *n* (%)	15,230 (28.46)	36,623 (22.83)	<0.001	15,230 (28.46)	14,270 (26.67)	<0.001
≥80, *n* (%)	33,213 (62.07)	82,146 (51.2)	<0.001	33,213 (62.07)	34,236 (63.99)	<0.001
CCI index, mean (SD)	1.28 (1.01)	0.99 (0.93)	<0.001	1.28 (1.01)	1.27 (1.00)	0.043
Myocardial infarction, *n* (%)	2583 (4.83)	3463 (2.16)	<0.001	2583 (4.83)	2415 (4.51)	0.015
Congestive heart failure, *n* (%)	18,422 (34.43)	36,101 (22.5)	<0.001	18,422 (34.43)	18,260 (34.13)	0.297
Peripheral vascular disease, *n* (%)	1789 (3.34)	3379 (2.11)	<0.001	1789 (3.34)	1934 (3.61)	0.016
Cerebrovascular disease, *n* (%)	4055 (7.58)	8021 (5)	<0.001	4055 (7.58)	3962 (7.4)	0.280
Dementia, *n* (%)	6875 (12.85)	17,334 (10.8)	<0.001	6875 (12.85)	6982 (13.05)	0.330
COPD, *n* (%)	11,595 (21.67)	36,351 (22.66)	<0.001	11,595 (21.67)	11,754 (21.97)	0.239
Rheumatoid disease, *n* (%)	1771 (3.31)	6204 (3.87)	<0.001	1771 (3.31)	1818 (3.4)	0.425
Peptic ulcer, *n* (%)	183 (0.34)	681 (0.42)	0.009	183 (0.34)	169 (0.32)	0.455
Mild liver disease, *n* (%)	2161 (4.04)	5570 (3.47)	<0.001	2161 (4.04)	2126 (3.97)	0.585
Hemiplegia or paraplegia, *n* (%)	367 (0.69)	1162 (0.72)	0.361	367 (0.69)	321 (0.6)	0.079
Renal disease, *n* (%)	14,464 (27.03)	22,520 (14.04)	<0.001	14,464 (27.03)	13,939 (26.05)	<0.001
Cancer, *n* (%)	2653 (4.96)	10,202 (6.36)	<0.001	2653 (4.96)	2653 (4.96)	0.999
Moderate/severe liver disease, *n* (%)	438 (0.82)	997 (0.62)	<0.001	438 (0.82)	434 (0.81)	0.892
Metastatic cancer, *n* (%)	1077 (2.01)	5599 (3.49)	<0.001	1077 (2.01)	1027 (1.92)	0.271
AIDS, *n* (%)	92 (0.17)	1380 (0.86)	<0.001	92 (0.17)	67 (0.13)	0.047
Non-invasive mechanical ventilation, *n* (%)	1754 (3.28)	3992 (2.49)	<0.001	1754 (3.28)	1263 (2.36)	<0.001
Invasive mechanical ventilation, *n* (%)	907 (1.70)	3503 (2.18)	<0.001	907 (1.70)	1027 (1.92)	0.006
LOHS, median (IQR)	7 (6)	7 (7)	0.555	7 (6)	7 (7)	0.737
IHM, *n* (%)	6908 (12.91)	19,128 (11.92)	<0.001	6908 (12.91)	7606 (14.22)	<0.001

T2DM: type 2 diabetes mellitus; CCI: Charlson comorbidity index; COPD: chronic obstructive pulmonary disease; AIDS: acquired immune deficiency syndrome; LOHS: length of hospital stay; IHM: in-hospital mortality.

**Table 3:** Distribution of study covariates and hospital outcomes of men with and without T2DM hospitalized with community-acquired pneumonia (CAP) in Spain (2016–2019), before and after propensity score matching (PSM).

	Before PSM			After PSM		
	T2DM	No T2DM	*p*-Value	T2DM	No T2DM	*p*-Value
*N* (incidence per 10,000 men)	86,904 (132.92)	219,886 (31.33)	<0.001			
Age, mean (SD)	76.68 (10.56)	72.09 (16.39)	<0.001	76.68 (10.56)	77.2 (10.83)	<0.001
18–49, *n* (%)	1291 (1.49)	24,852 (11.3)	<0.001	1291 (1.49)	1391 (1.6)	0.052
50–64, *n* (%)	10,124 (11.65)	36,585 (16.64)	<0.001	10,124 (11.65)	9796 (11.27)	0.014
65–79, *n* (%)	35,906 (41.32)	67,279 (30.6)	<0.001	35,906 (41.32)	33,841 (38.94)	<0.001
≥80, *n* (%)	39,583 (45.55)	91,170 (41.46)	<0.001	39,583 (45.55)	41,876 (48.19)	<0.001
CCI index, mean (SD)	1.57 (1.12)	1.24 (1.05)	<0.001	1.57 (1.12)	1.54 (1.11)	<0.001
Myocardial infarction, *n* (%)	7883 (9.07)	11,393 (5.18)	<0.001	7883 (9.07)	7578 (8.72)	0.010
Congestive heart failure, *n* (%)	23,302 (26.81)	39,443 (17.94)	<0.001	23,302 (26.81)	22,545 (25.94)	<0.001
Peripheral vascular disease, *n* (%)	8793 (10.12)	13,655 (6.21)	<0.001	8793 (10.12)	8674 (9.98)	0.342
Cerebrovascular disease, *n* (%)	7699 (8.86)	13,116 (5.96)	<0.001	7699 (8.86)	7669 (8.82)	0.800
Dementia, *n* (%)	6910 (7.95)	15,588 (7.09)	<0.001	6910 (7.95)	6930 (7.97)	0.859
COPD, *n* (%)	34,885 (40.14)	81,353 (37)	<0.001	34,885 (40.14)	35,252 (40.56)	0.073
Rheumatoid disease, *n* (%)	1635 (1.88)	3966 (1.8)	0.147	1635 (1.88)	1606 (1.85)	0.607
Peptic ulcer, *n* (%)	546 (0.63)	1561 (0.71)	0.014	546 (0.63)	529 (0.61)	0.603
Mild liver disease, *n* (%)	5219 (6.01)	12,927 (5.88)	0.181	5219 (6.01)	5118 (5.89)	0.306
Hemiplegia or paraplegia, *n* (%)	623 (0.72)	2157 (0.98)	<0.001	623 (0.72)	579 (0.67)	0.203
Renal disease, *n* (%)	24,541 (28.24)	34,534 (15.71)	<0.001	24,541 (28.24)	23,832 (27.42)	<0.001
Cancer, *n* (%)	8429 (9.7)	23,777 (10.81)	<0.001	8429 (9.7)	8526 (9.81)	0.433
Moderate/severe liver disease, *n* (%)	1229 (1.41)	2722 (1.24)	<0.001	1229 (1.41)	1133 (1.3)	0.047
Metastatic cancer, *n* (%)	4022 (4.63)	13,513 (6.15)	<0.001	4022 (4.63)	4045 (4.65)	0.793
AIDS, *n* (%)	303 (0.35)	3395 (1.54)	<0.001	303 (0.35)	246 (0.28)	0.015
Non-invasive mechanical ventilation, *n* (%)	2903 (3.34)	6172 (2.81)	<0.001	2903 (3.34)	2364 (2.72)	<0.001
Invasive mechanical ventilation, *n* (%)	2242 (2.58)	6849 (3.11)	<0.001	2242 (2.58)	2636 (3.03)	<0.001
LOHS, median (IQR)	7 (7)	7 (7)	0.743	7 (7)	7 (7)	0.856
IHM, *n* (%)	10,544 (12.13)	27,808(12.65)	<0.001	10,544 (12.13)	12,368 (14.23)	<0.001

T2DM: type 2 diabetes mellitus; CCI: Charlson comorbidity index; COPD: chronic obstructive pulmonary disease; AIDS: acquired immune deficiency syndrome; LOHS: length of hospital stay; IHM: in-hospital mortality.

**Table 4:** Distribution of study covariates and hospital outcomes of men and women with T2DM hospitalized with community-acquired pneumonia (CAP) in Spain (2016–2019), before and after propensity score matching (PSM).

	Before PSM			After PSM		
	T2DM MEN	T2DM WOMEN	*p*-Value	T2DM MEN	T2DM WOMEN	*p*-Value
*N* (incidence per 10,000 subjects)	86,904 (132.92)	53,506 (90.82)	<0.001			
Age, mean (SD)	76.68 (10.56)	80.18 (10.79)	<0.001	79.38 (9.83)	80.18 (10.79)	<0.001
18–49, *n* (%)	1291 (1.49)	643 (1.2)	<0.001	313 (0.58)	643 (1.2)	<0.001
50–64, *n* (%)	10,124 (11.65)	4420 (8.26)	<0.001	4614 (8.62)	4420 (8.26)	0.033
65–79, *n* (%)	35,906 (41.32)	15,230 (28.46)	<0.001	17,173 (32.1)	15,230 (28.46)	<0.001
≥80, *n* (%)	39,583 (45.55)	33,213 (62.07)	<0.001	31,406 (58.7)	33,213 (62.07)	<0.001
CCI index, mean (SD)	1.57 (1.12)	1.28 (1.01)	<0.001	1.22 (1.00)	1.28 (1.01)	<0.001
Myocardial infarction, *n* (%)	7883 (9.07)	2583 (4.83)	<0.001	2607 (4.87)	2583 (4.83)	0.733
Congestive heart failure, *n* (%)	23,302 (26.81)	18,422 (34.43)	<0.001	17,349 (32.42)	18,422 (34.43)	<0.001
Peripheral vascular disease, *n* (%)	8793 (10.12)	1789 (3.34)	<0.001	2769 (5.17)	1789 (3.34)	<0.001
Cerebrovascular disease, *n* (%)	7699 (8.86)	4055 (7.58)	<0.001	4202 (7.85)	4055 (7.58)	0.092
Dementia, *n* (%)	6910 (7.95)	6875 (12.85)	<0.001	5969 (11.16)	6875 (12.85)	<0.001
COPD, *n* (%)	34,885 (40.14)	11,595 (21.67)	<0.001	11,807 (22.07)	11,595 (21.67)	0.117
Rheumatoid disease, *n* (%)	1635 (1.88)	1771 (3.31)	<0.001	1415 (2.64)	1771 (3.31)	<0.001
Peptic ulcer, *n* (%)	546 (0.63)	183 (0.34)	<0.001	169 (0.32)	183 (0.34)	0.455
Mild liver disease, *n* (%)	5219 (6.01)	2161 (4.04)	<0.001	2128 (3.98)	2161 (4.04)	0.607
Hemiplegia or paraplegia, *n* (%)	623 (0.72)	367 (0.69)	0.500	407 (0.76)	367 (0.69)	0.149
Renal disease, *n* (%)	24,541 (28.24)	14,464 (27.03)	<0.001	14,492 (27.08)	14,464 (27.03)	0.847
Cancer, *n* (%)	8429 (9.7)	2653 (4.96)	<0.001	2525 (4.72)	2653 (4.96)	0.068
Moderate/severe liver disease, *n* (%)	1229 (1.41)	438 (0.82)	<0.001	439 (0.82)	438 (0.82)	0.973
Metastatic cancer, *n* (%)	4022 (4.63)	1077 (2.01)	<0.001	706 (1.32)	1077 (2.01)	<0.001
AIDS, *n* (%)	303 (0.35)	92 (0.17)	<0.001	102 (0.19)	92 (0.17)	0.472
Non-invasive mechanical ventilation, *n* (%)	2903 (3.34)	1754 (3.28)	0.526	1521 (2.84)	1754 (3.28)	<0.001
Invasive mechanical ventilation, *n* (%)	2242 (2.58)	907 (1.7)	<0.001	1121 (2.1)	907 (1.7)	<0.001
LOHS, median (IQR)	7 (7)	7 (6)	0.088	7 (7)	7 (6)	0.060
IHM, *n* (%)	10,544 (12.13)	6908 (12.91)	<0.001	7213 (13.48)	6908 (12.91)	0.006

T2DM: type 2 diabetes mellitus; CCI: Charlson comorbidity index; COPD: chronic obstructive pulmonary disease; AIDS: acquired immune deficiency syndrome; LOHS: length of hospital stay; IHM: in-hospital mortality.

**Table 5:** Multivariable analysis of factors associated with in-hospital mortality during admissions for community-acquired pneumonia (CAP) among T2DM patients according to sex.

	Men	Women	Both
	OR (95% CI)	OR (95% CI)	OR (95% CI)
18–49 years	1	1	1
50–64 years	1.75 (1.33–2.3)	1.38 (0.9–2.13)	1.54 (1.07–2.2)
65–79 years	2.55 (1.95–3.34)	2.52 (1.66–3.81)	2.91 (2.05–4.12)
≥80 years	5.01 (3.83–6.56)	5.49 (3.63–8.29)	6.12 (4.32–8.67)
Myocardial infarction	1.08 (1–1.16)	1.32 (1.18–1.47)	1.21 (1.12–1.31)
Congestive heart failure	1.25 (1.2–1.32)	1.2 (1.13–1.26)	1.21 (1.17–1.26)
Peripheral vascular disease	NS	1.19 (1.04–1.37)	1.17 (1.05–1.31)
Cerebrovascular disease	1.48 (1.39–1.59)	1.59 (1.46–1.73)	1.57 (1.48–1.67)
Dementia	2.13 (2–2.27)	2 (1.87–2.14)	2.06 (1.97–2.17)
COPD	0.68 (0.65-0.72)	0.64 (0.6–0.69)	0.69 (0.66–0.73)
Mild liver disease	NS	0.81 (0.7–0.95)	0.87 (0.78–0.96)
Hemiplegia or paraplegia	2.27 (1.87–2.77)	2.43 (1.91–3.1)	2.08 (1.75–2.48)
Renal disease	1.14 (1.09–1.19)	1.15 (1.09–1.22)	1.15 (1.1–1.2)
Cancer	1.95 (1.83–2.08)	1.81 (1.63–2.02)	1.79 (1.66–1.93)
Moderate/severe liver disease	2.67 (2.31–3.09)	2.06 (1.6–2.65)	2.51 (2.11–2.97)
Metastatic cancer	4.71 (4.36–5.1)	4.61 (3.99–5.32)	4.18 (3.75–4.66)
Non-invasive mechanical ventilation	2.59 (2.35–2.85)	2.45 (2.17–2.77)	2.47 (2.26–2.7)
Invasive mechanical ventilation	8.56 (7.79–9.41)	7.34 (6.3–8.56)	8.08 (7.31–8.93)
2017	0.99 (0.93–1.06)	0.96 (0.89–1.04)	0.97 (0.92–1.02)
2018	0.95 (0.9–1.01)	0.89 (0.83–0.96)	0.92 (0.88–0.97)
2019	0.91 (0.85–0.97)	0.86 (0.8–0.93)	0.87 (0.83–0.92)
Male sex			1.06 (1.02–1.1)

OR: Odds Ratios. COPD: chronic obstructive pulmonary disease. NS: not significant.

## Data Availability

According to the contract signed with the Spanish Ministry of Health and Social Services, which provided access to the databases from the Spanish National Hospital Database (Conjunto Mínimo Basico de Datos; CMBD), we cannot share the databases with any other investigator, and we have to destroy the databases once the investigation has concluded. Consequently, we cannot upload the databases to any public repository. However, any investigator can apply for access to the databases by filling out the questionnaire available at http://www.msssi.gob.es/estadEstudios/estadisticas/estadisticas/estMinisterio/SolicitudCMBDdocs/Formulario_Peticion_Datos_CMBD.pdf. All other relevant data are included in the paper.

## Supplementary Materials

The following are available online at https://www.mdpi.com/article/10.3390/jcm10173975/s1, Table S1. ICD-10 codes for diagnosis and therapeutic procedures and pressure ulcers used in this investigation, Table S2. Distribution of pneumonia pathogens in patients with and without T2DM hospitalized with community-acquired pneumonia (CAP) in Spain from 2016 to 2019, Table S3. Distribution of pneumonia pathogens in women and men with T2DM hospitalized with community-acquired pneumonia (CAP), in Spain (2016–2019), before and after propensity score matching, Table S4. Univariable analysis of factors associated with in-hospital mortality during admissions for community-acquired pneumonia (CAP), among T2DM patients according to sex.
