# Supplementary material for: Sex-Related Disparities in the Incidence and Outcomes of Community-Acquired Pneumonia among Type 2 Diabetes Patients: A Propensity Score-Matching Analysis Using the Spanish National Hospital Discharge Database for the Period 2016–2019
Source: J Clin Med. 2021 Sep 2;10(17):3975. doi: 10.3390/jcm10173975 (PMC8432254; doi:10.3390/jcm10173975)
Supplement: Supplementary file 1 [file jcm-10-03975-s001.zip › jcm-1303663-supplementary.pdf]

Table S1. ICD-10 codes for diagnosis and therapeutic procedures and pressure ulcers used in this investigation.

	**ICD-10 codes**
Community-acquired pneumonia *	J12 to J18 as primary diagnosis with a POA indicator of "Y".<br>J12 to J18 in any of the secondary diagnosis fields (2-20) and with a POA indicator of "Y".
No invasive mechanical ventilation	5A09357, 5A09457, 5A09557
Invasive mechanical ventilation	5A1945Z, 5A1955Z, 5A1935Z
Aspergillus	B44.9
Escherichia coli	J15.5
Haemophilus influenzae	J14
Klebsiella pneumoniae	J15
Legionella	A48.1
Non specified *Streptococcus*	J15.4
Other Gram negative bacteria	J15.6
Influenza virus	J09.X1, J10.00 J10.01, J10.08, J11.0, J11.00, J11.08
Other virus	J12.XX
Pseudomonas aeruginosa	J15.1
Staphylococcus aureus	J15.211 AND J15.212
Streptococcus pneumoniae	J13

*Each discharge diagnosis has a "Present on Admission (POA)" indicator assigned according to the ICD-10-CM Official Guidelines for Coding and Reporting (**https://icdlist.com/icd-10/guidelines/**). The reporting options and definitions for POA are "Y" (present at admission); "N" (not present at admission); "U" (lack documentation to determine presence at admission); "W" (provider is unable to clinically determine if the condition was present); and unreported/not used.

Table. S2. Distribution of pneumonia pathogens in patients with and without T2DM hospitalized with community-acquired pneumonia (CAP) in Spain from 2016 to 2019

		2016	2017	2018	2019	p-value
Aspergillus, n(%)	**T2DM**	19(0.07)	21(0.06)	25(0.06)	18(0.05)	0.751
	**No T2DM**	86(0.1)	93(0.1)	99(0.1)	85(0.09)	0.656
Escherichia coli, n(%)	**T2DM**	123(0.42)	134(0.39)	168(0.43)	163(0.43)	0.776
	**No T2DM**	391(0.46)	418(0.46)	440(0.43)	423(0.42)	0.568
Haemophilus influenzae, n(%)	**T2DM**	186(0.64)	255(0.74)	328(0.84)	285(0.75)	0.024
	**No T2DM**	644(0.75)	736(0.8)	897(0.87)	964(0.97)	<0.001
Influenza pneumoniae, n(%)	**T2DM**	795(2.73)	879(2.54)	1830(4.7)	1633(4.32)	<0.001
	**No T2DM**	3321(3.88)	2504(2.73)	5434(5.26)	5074(5.1)	<0.001
Klebsiella pneumoniae, n(%)	**T2DM**	150(0.51)	159(0.46)	203(0.52)	196(0.52)	0.615
	**No T2DM**	377(0.44)	474(0.52)	482(0.47)	545(0.55)	0.004
Legionella, n(%)	**T2DM**	12(0.04)	20(0.06)	29(0.07)	22(0.06)	0.368
	**No T2DM**	47(0.05)	58(0.06)	90(0.09)	65(0.07)	0.044
Non specified *Streptococcus*, n(%)	**T2DM**	66(0.23)	77(0.22)	78(0.2)	105(0.28)	0.151
	**No T2DM**	224(0.26)	245(0.27)	327(0.32)	299(0.3)	0.073
Other Gram-negative bacteria, n(%)	**T2DM**	152(0.52)	190(0.55)	187(0.48)	250(0.66)	0.006
	**No T2DM**	528(0.62)	550(0.6)	580(0.56)	617(0.62)	0.309
Other virus, n (%)	**T2DM**	175(0.6)	220(0.64)	311(0.8)	425(1.13)	<0.001
	**No T2DM**	696(0.81)	674(0.73)	1061(1.03)	1185(1.19)	<0.001
Pseudomonas aeruginosa, n(%)	**T2DM**	309(1.06)	294(0.85)	339(0.87)	362(0.96)	0.022
	**No T2DM**	960(1.12)	1022(1.11)	1105(1.07)	1083(1.09)	0.690
Staphylococcus aureus, n(%)	**T2DM**	230(0.79)	299(0.87)	370(0.95)	342(0.91)	0.154
	**No T2DM**	725(0.85)	724(0.79)	870(0.84)	904(0.91)	0.044
Streptococcus pneumoniae, n(%)	**T2DM**	1915(6.57)	2542(7.35)	3396(8.72)	3851(10.2)	<0.001
	**No T2DM**	6584(7.69)	8008(8.72)	10724(10.38)	11402(11.45)	<0.001

T2DM: Type 2 diabetes mellitus;

Table. S3. Distribution of pneumonia pathogens in women and men with T2DM hospitalized with community-acquired pneumonia (CAP), in Spain (2016-2019), before and after propensity score matching.

	Before PSM			After PSM		
	**T2DM Men**	**T2DM Women**	**p-value**	**T2DM Men**	**T2DM Women**	**p-value**
Aspergillus, n(%)	57(0.07)	26(0.05)	0.203	24(0.04)	26(0.05)	0.777
Escherichia coli, n(%)	430(0.49)	158(0.3)	<0.001	218(0.41)	158(0.3)	0.002
Haemophilus influenzae, n(%)	719(0.83)	335(0.63)	<0.001	361(0.67)	335(0.63)	0.323
Influenza pneumoniae, n(%)	2948(3.39)	2189(4.09)	<0.001	1901(3.55)	2189(4.09)	<0.001
Klebsiella pneumoniae, n(%)	509(0.59)	199(0.37)	<0.001	261(0.49)	199(0.37)	0.004
Legionella, n(%)	65(0.07)	18(0.03)	0.002	45(0.08)	18(0.03)	0.001
Non specified *Streptococcus*, n(%)	247(0.28)	79(0.15)	<0.001	140(0.26)	79(0.15)	<0.001
Other Gram-negative bacteria, n(%)	589(0.68)	190(0.36)	<0.001	286(0.53)	190(0.36)	<0.001
Other virus, n(%)	609(0.7)	522(0.98)	<0.001	374(0.7)	522(0.98)	<0.001
Pseudomonas aeruginosa, n(%)	959(1.1)	345(0.64)	<0.001	414(0.77)	345(0.64)	0.012
Staphylococcus aureus, n(%)	856(0.98)	385(0.72)	<0.001	478(0.89)	385(0.72)	0.001
Streptococcus pneumoniae, n(%)	7311(8.41)	4393(8.21)	0.183	4466(8.35)	4393(8.21)	0.418

Table S4. Univariable analysis of factors associated with in-hospital mortality during admissions for community-acquired pneumonia (CAP), among T2DM patients according to sex.

	**MEN**	**WOMEN**	**BOTH**
Variables	**OR (95%CI)**	**OR (95%CI)**	**OR (95%CI)**
18-49 years	1	1	1
50-64 years	1.82(1.40-2.25)	1.49(1.08-2.12)	1.60(1.12-2.31)
65-79 years	2.97(2.33-3.59)	3.03(2.14-4.43)	3.14(2.19-4.34)
≥ 80 years	5.54(4.25-7.12)	7.28(5.71-9.34)	6.89(4.76-9.05)
Myocardial infarction	1.23(1.11-1.37)	1.59(1.42-1.78)	1.47 (1-29-1.56)
Congestive heart failure	1.42(1.36-1.55)	1.60(1.51-1.70)	1.53 (1.32-1.61)
Peripheral vascular disease	1.20(1.06-1.37)	1.37(1.21-1.54)	1.29(1.18-1.42)
Cerebrovascular disease	1.58(1.47-1.70)	1.69(1.51-1.89)	1.63(1.51-1.76)
Dementia	2.18(2.07.2.20)	1.97(1.82-2.14)	1.97(1.80-2.15)
COPD	0.91(0.84-0.98)	0.88(0.82-0.96)	0.83(0.78-0.89)
Rheumatoid disease	1.17(1.01-1.35)	1.10(0.94-1.28)	1.12(0.97-1.26)
Peptic ulcer	0.88(0.75-1.03)	1.02(0.84-1.23)	0.97(0.81-1.15)
Mild liver disease	0.91(0.86-0.99)	0.83(0.74-0.93)	0.90(0.79-1.01)
Hemiplegia or paraplegia	2.37(1.92-2.83)	2.51(1.95-3.27)	2.39(2.01-2.80)
Renal disease	1.26(1.20-1.32)	1.56(1.47-1.66)	1.37(1.30-1.45)
Cancer	2.16(2.01-2.34)	1.99(1.83-2.16)	2.05(1.90-2-22)
Moderate/severe liver disease,	3.46(3.01-3.99)	2.88(2.35-3.43)	3.04 (2.63-3.46)
Metastatic cancer	6.37(5.87-6.88)	5.89(5.12-6.79)	6.12(5.69-6.57)
AIDS	2.02(0.97-3.08)	1.25(0.79-1.77)	1.80(0.72-2.89)
Non-Invasive Mechanical ventilation,	3.33(3.01-3.65)	2.84(2.49-3.27)	3.23(2.98-3.50)
Invasive Mechanical ventilation	11.12(8.99-13.34)	9.16(8.03-10.19)	10.11(9.23-12.17)
2017	0.93(0.86-0.99)	0.92(0.85-0.99)	0.90(0.84-0.98)
2018	0.90(0.84-0.97)	0.84(0.79-0.91)	0.85(0.80-0.91)
2019	0.87(0.81-0.94)	0.80(0.72-0.89)	0.82(0.75-0.90)
Male sex			1.12 (1.07-1.17)

OR: Odds Ratios. T2DM: Type 2 diabetes mellitus; COPD: chronic obstructive pulmonary disease; AIDS: acquired immune deficiency syndrome
